# The Biofeedback Therapy in Patients With Stress Incontinence Urinary

**DOI:** 10.5812/numonthly.4153

**Published:** 2012-06-20

**Authors:** Maria Thereza Micussi

**Affiliations:** 1 Health Science Center, UFRN, Natal, Rio Grande do Norte, Brazil

**Keywords:** Therapeutics, Urinary Incontinence

Dear Editor,

I read with great interest the article by Seckiner *et al.* entitled “The Effect of Biofeedback Therapy on ICIQ-SF Scores and Urodynamic Parameters in Patients with Stress Urinary Incontinence” (1). The authors concluded that biofeedback therapy is capable of decreasing ICIQ-SF scores, that is, therapy improves symptoms and the impact of urinary incontinence on the patients’ quality of life. Dumoulin *et al.* (2) showed that training pelvic floor muscles is effective in treating stress urinary incontinence (SUI) and this can be optimized when combined with biofeedback. On the other hand, Herdeschee et al. (3) suggested that women receiving biofeedback therapy are more likely to report that their urine loss was cured or improved, when compared to a group of women who had only undergone muscle training (RR = 0.75). Furthermore, in the authors’ opinion, a urodynamic test is not necessary for patients who have submitted to biofeedback treatment. Although the gold standard for assessing SUI is a urodynamic examination, in addition to being costly, it is not well accepted by patients (4). Other methods, such as the pad test, are more accessible and have been used in clinical practice to assess, classify and quantify urinary losses (5, 6, 7). The 1-hour version is valid and recommended by the International Continence Society (8). Results show high specificity and sensitivity when compared with a urodynamic examination (9).

Another aspect discussed in the article is the need for exercise continuity at home. I am in total agreement with the authors, since exercises must be continued in order to avoid the emergence or worsening of symptoms. This matter should be emphasized more in studies, and include a description of the forms of patient adherence and incentives, especially over the long term.
